# Perceived Barriers and Facilitators Regarding the Implementation of Gamification to Promote Physical Activity in the Neighborhood: Interview Study Among Intermediaries

**DOI:** 10.2196/52991

**Published:** 2024-08-28

**Authors:** Ayla Schwarz, Kirsten Verkooijen, Emely de Vet, Monique Simons

**Affiliations:** 1 Wageningen University & Research Wageningen Netherlands; 2 Tilburg School of Humanities and Digital Sciences University College Tilburg Tilburg Netherlands

**Keywords:** gamification, gamified apps, physical activity, implementation, intermediaries, interview

## Abstract

**Background:**

In the Netherlands, neighborhood sport coaches (NSCs) play an important role as intermediaries in promoting physical activity (PA) in the neighborhood. Gamification is the use of game elements in nongame contexts; it can be implemented with or without technology and holds promise for promoting PA. NSCs infrequently make use of this option.

**Objective:**

This study aims to understand barriers to, and facilitators of, using gamification to promote PA, as perceived by NSCs.

**Methods:**

A total of 25 semistructured interviews were conducted with NSCs in the Netherlands. The interviews were audiotaped, transcribed, and analyzed by means of thematic analysis using ATLAS.ti (version 22; ATLAS.ti Scientific Software Development GmbH) software. The deductive coding was informed by the capability, opportunity, motivation, behavior model and the theoretical domains framework, complemented by inductive coding.

**Results:**

Barriers and facilitators identified as factors influencing the implementation of gamification were related to 7 themes. NSCs required technical, creative, and promotion skills; knowledge about existing gamification tools; and social support from their employer and professional network. Financial costs were identified as a barrier to the successful implementation of gamification. Lack of clarity regarding stakeholders’ responsibility to implement gamification could further hamper implementation. In general, NSCs were positive about investing time in implementing gamification and expected positive effects from implementing it.

**Conclusions:**

To overcome identified barriers, a clear overview of tools, best practices, and available subsidies must be created, a gamification network must be established, the responsibility of NSCs must be clarified, and guidance must be offered on the promotion of gamification.

## Introduction

### Background

Physical activity (PA) supports both physical and mental health. It can prevent noncommunicable diseases, such as cardiovascular diseases, type 2 diabetes, hypertension, and certain types of cancer [1-3], and it reduces the risk of it reduces the risk of becoming overweight or developing obesity [4]. However, worldwide PA prevalence is low, especially in high-income countries [5]. PA guidelines for children recommend daily moderate to vigorous PA for 60 minutes and vigorous activities 3 days per week. PA guidelines for adults recommend at least 150 minutes of moderate-intensity aerobic PA each week, a minimum of 75 minutes of vigorous-intensity aerobic PA or an equivalent combination of both. Muscle-strengthening activities involving major muscle groups should be performed at least 2 days a week [6]. In the Netherlands, 43% of children (aged 4-11 years), 62% of youths (aged 12-17 years), and 54% (aged 18-64 years) of adults did not meet the PA recommendations in 2022 [7]. Therefore, PA interventions are needed to increase PA levels in all populations.

Intermediaries or health brokers play an important role in offering accessible (ie, geographically close and inexpensive) PA interventions that are tailored to local communities’ needs and wishes [8,9]. In the Netherlands, the Dutch Ministry of Health, Welfare, and Sport introduced neighborhood sport coaches (NSCs; in Dutch *buurtsportcoach*) with the aim of connecting the sport sector with other sectors (eg, daycare and welfare) and of promoting PA and sports participation in the neighborhood. NSCs are responsible for supporting local sports clubs, promoting a specific sport (eg, hockey or soccer), or promoting informal PA (eg, physical exertion such as running, jumping, and dancing). These coaches are 40% funded by the state and 60% funded by the municipality or other local organizations. Currently, NSCs are strategically part of the local policy of almost all municipalities in the Netherlands [10]. In each municipality, the NSC’s role is unique, thereby allowing municipalities to implement activities in line with local needs and contexts. Examples of activities organized by NSCs include sports clinics, PAs during or after school, and sports tournaments. The latest trend, gamification, is increasingly gaining popularity and interest among NSCs [11,12] and is increasingly applied in PA interventions [13].

Gamification is the use of game elements in nongame contexts [14]. It draws from various theories, with the self-determination theory being the most frequently applied [15], particularly in PA interventions [13]. Self-determination theory supports the proposition that games and gamification can intrinsically motivate individuals to initiate and sustain health behaviors [16] by leveraging people’s natural desires for autonomy, competence, and relatedness [17]. Gamification is a set of motivational techniques that can enhance long-term engagement with apps [18], and it offers a promising route for behavioral change [17,19]. It can be implemented either technologically or nontechnologically. Examples of nontechnological gamification include points, badges, challenges, or leaderboards. Technological implementations include, for example, urban dance grounds, digital game walls or playgrounds, or gamified apps. These gamified apps apply gamified elements to enhance user engagement and enjoyment, as seen in popular apps such as Strava (Strava, Inc), Geocaching (Groundspeak, Inc), or Pokémon Go (Niantic, Inc). Gamification is most often applied in PA interventions, and systematic reviews and meta-analyses indicate that it has small to medium effects on various PA outcomes [20-22]. Increased use of gamification elements was associated with greater effectiveness in promoting moderate to vigorous PA [18]. These gamified PA interventions have been shown to affect PA behavior in a broad range of populations, regardless of age or health status [13,18,20], and to be more effective compared with equivalent nongamified interventions [13,18]. Johnson et al [20] identified 7 promises of gamification: enhancing intrinsic motivation, broad accessibility, broad appeal, broad applicability, cost-benefit efficiency, everyday-life fit, and supporting well-being. These benefits of gamification [13,20] align well with NSCs’ function of aligning their activities with the local population’s needs, ensuring the everyday-life fit of their activities, engaging a wider population, and supporting well-being. Therefore, implementing gamification through NSCs may hold promise.

Despite these benefits and the availability of gamification tools such as interactive (outdoor) play equipment (eg, interactive game walls) or gamified apps (eg, Seppo [Seppo.io]), NSCs’ actual use of gamification appears to remain low (Schwarz, AF, unpublished data, December 2022). Insight into factors underlying of implementation of gamification is needed to determine whether gamification aligns with NSCs’ intentions and, if so, to better understand how to encourage NSCs to implement gamification. Offering gamification via intermediaries (eg, health professionals, NSCs, and health brokers) has been barely researched [23]. Instead, gamification research has so far focused largely on the development of gamified interventions from end users’ perspectives and on the interventions’ effectiveness on behavior change in end users [24]. Consequently, knowledge on the strategic implementation of gamified interventions at a systemic level is still lacking, despite this being invaluable for a successful and sustainable uptake of innovations [24].

### This Study

Implementation science, which comprises the study of methods to promote the translation and uptake of evidence-based research into practice, has grown as a research field in the last decades. The aim of implementation science is 2-fold. On one hand, it aims to identify barriers to, and facilitators of, uptake. On the other hand, it aims to develop and apply implementation strategies to overcome identified barriers and enhance facilitators in the adoption of innovations [25]. Various implementation frameworks, such as the capability, opportunity, motivation, behavior (COM-B) model and the theoretical domains framework (TDF), have been developed to identify and understand barriers and facilitators [26,27]. Both frameworks can be classified as determinant frameworks that aim to understand influences, including barriers to, and facilitators of, implementation outcomes. This study applied both COM-B and TDF to better understand the perceived barriers to, and facilitators of, intermediaries’ gamification implementation, a topic that is barely researched.

## Methods

### Overview

This research is part of a larger project called mVital@2040 [28], which investigates gamification to promote PA in the neighborhood among youth (aged 10-14 years) living in low-income neighborhoods. In the first step, the project aimed to investigate how NSCs can implement gamification. For this study, we conducted semistructured interviews with NSCs in 5 municipalities in the Netherlands. These 5 municipalities, ranging from small (approximately 55,000 inhabitants) to large (>250,000 inhabitants), had 56,000; 238,000; 244,000; 368,000; and 918,000 inhabitants, respectively.

### Ethical Considerations

All NSCs provided informed consent with the option to opt out, and the study was approved by the Social Sciences Ethics Committee of Wageningen University & Research on March 23, 2022 (2022-45-Schwarz). NSCs did not receive any compensation for their participation, and all personally identifiable information was pseudonymized. Informed consent was obtained by accepting the Microsoft Teams invitation. At the start of each interview, the researcher once again asked for verbal informed consent.

### Selection and Study Population

For the purpose of this study, 6 intermediary organizations (eg, youth club and sports club) that employed NSCs were contacted across 5 municipalities in the Netherlands. At each organization, 1 contact person made a preselection of available NSCs, including NSCs both with and without prior experience with gamification or gamified apps to research experienced and expected barriers to, and facilitators of, gamification implementation. All age ranges of NSCs’ target group, ranging from young children to older persons, were included. Preselected candidates were invited by email to take part in an interview. A total of 31 NSCs were contacted, and 25 (81%) interviews were held.

### Procedure

The interviews were held between April and September 2022. The interviews took place via Microsoft Teams and lasted approximately 1 hour. All interviews were conducted by 1 researcher (AS).

The interview guide can be found in Multimedia Appendix 1. Topics discussed were related to NSCs’ function profile, the target group that they aim to reach, their definition of gamification, the role of gamification in their work, inhibiting and promoting factors regarding gamification implementation, and the envisioned future of gamification. The interview guide was informed by the COM-B model [29]. The model proposes that behavior arises from a result of the interaction among 3 components: capability (ie, people must have the knowledge, skills, and strength to perform the behavior), opportunity (ie, the physical and social environment must enable the behavior to occur, by, eg, being accessible, affordable, or acceptable), and motivation (ie, people must be motivated to perform the relevant time). Although best known for its application to understand behavior change in the receivers of health interventions, COM-B has been deemed useful to focus on professionals that incorporate health interventions targeted at a variety of audiences [30-32]. Designing the interview guide based on the COM-B model allowed us to create open questions to identify perceived barriers and facilitators.

### Data Analysis

The interviews were audiotaped and transcribed verbatim by an external company. The researcher who conducted the interviews checked the transcripts for accuracy. The transcripts were coded and analyzed using ATLAS.ti (version 22; ATLAS.ti Scientific Software Development GmbH) software. Data were inductively and deductively coded [33] by 1 researcher (AS) and double-coded by a second researcher (KV). Researchers compared the deductive part of the coding until a consensus was reached. In total, 3 (12%) interviews were double-coded.

Deductive coding was based on the COM-B [29] model and TDF [34] (Table 1). Both models allow the identification of barriers and facilitators influencing the uptake and use of gamification. COM-B maps all barriers and facilitators in a simple model of behavior, and TDF complements COM-B with a detailed outline of psychological capability and reflective motivation [34]. TDF contains 14 domains, derived from 33 theories and 128 psychological constructs. These can be mapped onto the components of the COM-B model [29]. The data analysis was based on thematic analysis [35], shifting from codes (TDF) to themes, which are patterns deriving from the large sample of inductive and deductive codes. In the Results section, nonspecific terms of semiquantification (eg, few, several, some, or many) are used to convey general patterns within the data [36].

**Table 1:** COM-B^a^ and TDF^b^ as outlined in the study by Michie et al [29].

COM-B component and domains (TDF)		Definition
**Physical capability**		
	Physical skills	An ability or proficiency acquired through practice
**Psychological capability**		
	Knowledge	An awareness of the existence of something
	Cognitive and interpersonal skills	An ability or proficiency acquired through practice
	Memory, attention, and decision processes	The ability to retain information, focus selectively on aspects of the environment, and choose between ≥2 alternatives
	Behavioral regulation	Anything aimed at managing or changing objectively observed or measured actions
**Physical opportunity **		
	Environmental context and resources	Any circumstance in a person’s situation or environment that discourages or encourages the development of skills and abilities, independence, social competence, and adaptive behavior
**Social opportunity **		
	Social influences	Those interpersonal processes that can cause individuals to change their thoughts, feelings, or behaviors
**Automatic motivation**		
	Emotions	A complex reaction pattern, involving experiential, behavioral, and physiological elements, by which the individual attempts to deal with a personally significant matter or event
	Reinforcement	Increasing the probability of a response by arranging a dependent relationship, or contingency, between the response and a given stimulus
**Reflective motivation**		
	Social and professional role and identity	A coherent set of behaviors and displayed personal qualities of an individual in a social or work setting
	Beliefs about capabilities	Acceptance of the truth, reality, or validity of an ability, talent, or facility that a person can put to constructive use
	Optimism	The confidence that things will happen for the best or that desired goals will be attained
	Intentions	A conscious decision to perform a behavior or a resolve to act in a certain way
	Goals	Mental representations of outcomes or end states that an individual wants to achieve
	Beliefs about consequences	Acceptance of the truth, reality, or validity of outcomes of a behavior in a given situation

^a^COM-B: capability, opportunity, motivation, behavior.

^b^TDF: theoretical domains framework.

## Results

### NSCs’ Work Profile

We interviewed 25 NSCs from 5 municipalities in the Netherlands. The spread of participants (4, 8, 8, 4, 1) differed depending on the size of the municipality. Of these 25 NSCs, 18 (72%) indicated that they had worked <5 years at their organization, representing the level of work experience. NSC teams typically consisted of 1 coordinating NSC that supervised multiple executive NSCs. The NSCs had predominately an executive role (17/25, 68%), including tasks such as organizing sports activities and events in the neighborhood (eg, at schools or public parks) and recruiting members of the target group for activities and events. The other 8 (32%) NSCs had a coordination role, consisting of project planning, coordinating, and connecting different organizations and parties in the neighborhood, and supporting executive NSCs. Responsible roles and tasks differed between municipalities, and not all municipalities differentiated between executive and coordinating NSCs.

### NSCs’ Target Groups

Many NSCs focused on a predefined age group (eg, children aged 6-12 years, youth, children and adults, and older persons), which was determined by the responsible municipality or organization. Some, however, indicated that they were responsible for all age groups. NSCs affirmed that they had perceived a shift over the last few years from a focus on younger children (eg, aged 6-12 years) to adolescents (aged 12-16 years). Besides specific age groups, NSCs targeted people who were physically inactive or people in a low socioeconomic position. NSCs focused on hard-to-reach groups, such as adolescents (especially female adolescents), children who were physically inactive, people with a low socioeconomic position or multiple problems or constraints, people with a language barrier, young adults with depression, older people, and young adult refugees.

### Level of Experience With Gamification

Quotes relating to the questions posed to NSCs are provided in Textbox 1. Overall, 20 (80%) of the 25 NSCs had some experience with gamification in general, and gamification was integrated into their program more naturally (quote 1, Textbox 1). In general, NSCs wanted to learn more about the possibility of applying gamified apps and were positive about committing themselves to adopting gamification but were still looking to see how they could apply it in practice (quote 2, Textbox 1). Examples of current applications of gamification were (1) translating web-based games (eg, Fortnite [Epic Games, Inc]) to real-life activities; (2) adding points systems, competition, and prize-winning to their current activities (eg, photo challenges and social media leaderboards); (3) implementing smart devices, such as light poles (eg, Interactive Play System cones changing colors) or smart walls; (4) reinventing a common sport (eg, James Bond game); and (5) gamified bicycle routes. Many NSCs reported a lack of experience with gamified apps. Those who had some experience implemented gamified apps sporadically. Examples of gamified apps were (1) popular social and dance-related media apps (eg, TikTok [ByteDance] and Just Dance [Ubisoft Inc]), (2) apps particularly designed to increase PA (eg, Missie Master [8D Games BV], Fitcoins [It’s My Life–Corporate & Individual well-being BV], Strava [Strava Inc], City Legends [CityLegends BV], Jachtseizoen [StukTV], and VR game), or (3) apps designed by the employer to link a reward system to their current PA offer.

**Numbered quotes, listed per theme level of experience with gamification, defining gamification, skills, and knowledge.**

**Level of experience with gamification**

- Quote 1: “I think [gamification] comes back, in a light form in a lot of activities in our work as [NSCs], making an activity playful.” [Executive neighborhood sport coach; NSC]
- Quote 2: “I would like to broaden my er, my world. Ehm, with regard to app use.... I think there is much more to be achieved than we as an organization are currently doing.” [Executive NSC]

**Defining gamification**

- Quote 3: “You have certain game elements...that you have in games, such as competition, leaderboards...and translate that into our own sports-promotion activities. That can be done in many ways...by means of apps, but that can also be done by being critical: well, you have sports equipment...a traditional football and a volleyball, but now there are also a lot of exercise products that are completely separate from a certain sport with the main aim of being fun.... For me, it is actually a very broad term, but to summarize it is about translating game elements elsewhere to that traditional sports world.” [Executive NSC]

**Skills**

- Quote 4: “I’m not constantly thinking about [designing new content for the app]. So that’s quite a shame, eh, that we don’t all have that [creative skill] from ourselves, but at the same time I don’t think you can eh, force something like that. You have to have a little, er, [technical] feeling and I just have that a little less than my colleagues.” [Executive NSC]
- Quote 5: “One says, you have to start small.... One of our [executive NSCs] used to promise a lot and was very enthusiastic and creative, but, in the end, they do not manage in ‘how are we going to apply that in a good way?’ And, in the end, they get frustrated because it doesn’t work, so that’s from a practice point-of-view.” [Coordinating NSC]
- Quote 6: “This is [an effective] tool, at least if promotion eh, support from the municipalities and other parties, when this is assured, then this could be the tool.” [Executive NSC]
- Quote 7: “It should actually be ensured that it will just become something normal. And it shouldn’t be that they do it because it’s new and then they um, again um, ignore it because um, the novelty wears off. So, it has to – they have to be stimulated every time, uh, to use the app, I think.” [Executive NSC]

**Knowledge**

- Quote 8: “In our work it is difficult to determine first, what exactly do we want, where, in which direction do we want to go, do we have enough evidence for this, do we have to find out whether this is already happening elsewhere, in other municipalities or cities, what are their experiences with this and then we already notice that we have lost quite a bit of time just figuring that out before anything really gets off the ground.” [Coordinating NSC]

### Defining Gamification

The definition of gamification varied among NSCs, and, at the onset of the interview, not all NSCs (8/25, 32%) were acquainted with the term. Many NSCs stated that they found it difficult to define gamification. They defined gamification mostly in terms of applying game elements, either on the web or offline, with the overall aim of promoting behavioral change. Others defined it in terms of the direct translation of web-based games to offline activities (eg, playing Super Mario [Nintendo] in real life), the application of different game elements (eg, competition or leaderboards) to offline traditional sport activities, or playful learning (quote 3, Textbox 1). Some NSCs defined gamification as the trend of increasingly making use of digital games or being increasingly more on the web and stated that they were especially confused by multiple terms, such as gamification, gaming, and esports used interchangeably.

### Barriers to, and Facilitators of, Implementing Gamification

#### Overview

NSCs made a distinction between implementing gamification during their program (eg, integrating gamification on the site) or offering it as an extension to their program (eg, offering a gamified app during holidays, when NSCs are not physically present). In addition, 7 topics relating to barriers to, and facilitators of, gamification implementation were identified, namely, skills, knowledge, expected effects, responsibility, social support, financial costs, and time. Barriers and facilitators were discussed equally often, except for expected effects and responsibility, which were raised more often.

#### Skills

NSCs with gamification experiences felt very capable of applying this to their program compared with NSCs with no former experience. They felt more capable of applying gamification tools, which they often already applied unconsciously and did not require technical skills. Creative skills also appeared necessary. Some gamified apps offered the possibility to ideate and create new content, for example, challenges. Although this feature supported NSCs to use apps, it sometimes also impeded structural implementation. NSCs noticed that they continuously needed to create new content to keep the app up-to-date. Thinking continuously about new content was considered tiresome and could, together with a lack of creativity to ideate new content, hinder the implementation of a gamified app (quote 4, Textbox 1).

The development of their creative skills was influenced by starting with small steps and their former education (quote 5, Textbox 1). Several requirements need to be met to use the potential of gamification fully, such as a successful promotion of gamified apps (quote 6, Textbox 1). NSCs pointed out that they lacked skills in promoting gamification tools among the target group. In some cases, their employer took over the promotion (eg, public relations department), yet, neither succeeded in attracting (app) users (long term), which eventually resulted in the decision to stop offering a gamified app. Moreover, NSCs confirmed that it was a challenge to keep the target group engaged, especially when the novelty effect wore off (quote 7, Textbox 1).

#### Knowledge

NSCs indicated that their knowledge of gamification was limited, as keeping track of all trends demanded a lot of time. An overload of apps in different app stores hampered the search for suitable gamified apps. A clear overview of gamification tools (eg, examples from other municipalities), in addition to workshops, would help NSCs acquire new knowledge. Proven effects and shared experiences contributed to the decision to implement gamification (quote 8, Textbox 1).

#### Expected Effects

NSCs generally believed in the positive effects of gamification. Contributory factors included their own experiences, scientific evidence on the effectiveness of gamification, and the target group experiencing it as fun. However, not having an affinity for gamification, irrespective of experience, hampered implementation (quote 9, Textbox 2). NSCs stated that gamification could contribute to lowering the threshold to engage in their program, to reaching a broad target group that is often perceived as difficult to reach (eg, people who were inactive), and to reaching people from a distance (eg, during holidays). Further, gamification was considered easy to organize, and gamified apps were appreciated in terms of having one place where all information could be bundled, and valuable insights could be found based on user data. However, NSCs also noticed risks with regard to gamified apps. They feared that the target group’s (especially children’s) total screen time would increase and that the target group would be distracted by other apps or notifications on their phone while using the intended gamified app, and therefore NSCs feared that they would contribute to the digital divide (eg, by excluding people who have no phone or speak another language).

**Numbered quotes, listed per theme expected effects and responsibility.**

**Expected effects**

- Quote 9: “I don’t have anything with that at all, not myself, not personally. So um, I’m not into eh, iPads or computers. I don’t have anything with that at all. So – I don’t like it anyway.” [Coordinating neighborhood sport coach; NSC]

**Responsibility**

- Quote 10: “I also think that as part of the role as NSC we should simply keep up with the times and this is no longer just the standard games as before. But we, uh, have to go along with it.” [Executive NSC]
- Quote 11: “I like to move with the times...then I’d rather be too early with something than...too late.” [Executive NSC]
- Quote 12: “Um, the question is, whose task is it? Maybe of the municipality? or education? Ehm, and I think that er, that issue has never actually been mentioned and as long as, as long as no one actually raises it, it will actually not be taken up, eh, I think.” [Coordinating NSC]
- Quote 13: “To share the knowledge, that would be my task.... So, I should actually acquire the knowledge first and then share it among our [executive NSCs]. So, it should be the responsibility of the [coordinating NSCs] to train.” [Coordinating NSC]
- Quote 14: “The [executive NSCs] we employ are responsible for one sport. And they don’t yet have enough knowledge to continue with the bigger piece. And they are used to just providing fun sports lessons and being enthusiastic about their sport. But [gamification] requires something completely different – that has a completely different goal than if you try to get the kids excited to start playing [one particular] sports and let them discover what they like .... So, that’s quite a big shift for them. So, it requires a different way of looking at how you organize your lessons and a different way of looking at the children you have in your group and that takes quite a lot.” [Coordinating NSC]
- Quote 15: “Sometimes you just need to be handed the [gamification] solution to eventually do it.” [Executive NSC]
- Quote 16: “Eh, if this is an assignment from the municipality, eh, then I feel that we should just, eh, take it up together.” [Executive NSC]
- Quote 17: “Who takes care of it and is also going to implement it and, er, everything that comes with it. So, the implementation of the lessons, the implementation of new elements eh, having knowledge about it, but also the communication of, okay, how are we going to promote it?” [Coordinating NSC]
- Quote 18: “It is good that a number of colleagues are in the lead and are really going to pick that up and roll it out and then maybe give me a concrete assignment of, you now have to come up with a mission and then I will think about it. But I don’t quickly start from myself.” [Executive NSC]

#### Responsibility

NSCs viewed the adoption of gamification in their professional responsibilities as a means of keeping up with the latest developments and trends (quote 10 and Q11, Textbox 2). At the same time, NSCs questioned whose responsibility it was to adopt gamification eventually (quote 12, Textbox 2). One coordinating NSC considered knowledge transfer as part of their responsibility (quote 13, Textbox 2). Another coordinating NSC indicated that imposing the responsibility on executive NSCs could be challenging (quote 14, Textbox 2). Appointing a responsible NSC in the team and receiving a direct assignment by the supervisor or policy makers were deemed to promote implementation (quotes 15 and 16, Textbox 2). Yet, many NSCs indicated that it was difficult to find suitable, but indispensable, persons to enthusiastically promote gamification in the field (quote 17, Textbox 2). Reinforcement by colleagues or supervisors was considered essential to keep reminding them to ideate and create new content or updates for a gamified app. A clear assignment by supervisors can support NSCs. However, once they noticed that the gamified app was not or barely used, they felt demotivated to create new content or to promote the gamified app. NSCs clearly designated reinforcement as a responsibility of coordinating NSCs (quote 18, Textbox 2).

#### Social Support

NSCs received both direct support from their employer and support from external parties, such as schools, municipalities, or commercial sports clubs.

#### Support by Employer

Overall, NSCs emphasized that they experienced a lot of freedom in performing their job. Freedom was allowed, as long as activities aligned with national or local sports agreements that often reflected the needs of the neighborhood for which they worked (quote 19, Textbox 3). NSCs felt supported by their employer to apply gamification (quote 20, Textbox 3). However, several NSCs asserted that they did not receive active support to apply gamification. They often used social media to get inspiration on how to apply gamification. Sharing this content with their direct colleagues contributed to the implementation of gamification tools.

**Numbered quotes, listed per theme social support, external support, financial costs, and time.**

**Social support**

- Quote 19: “I do have a lot of freedom and that stems, I think, from the vision of, er, our employer, that, er, every neighborhood actually needs customization. So, what works in my neighborhood may not work in another neighborhood for example. So, it is also necessary to have some freedom, to be able to test some things and then also build on that.” [Executive neighborhood sport coach; NSC]
- Quote 20: “I just notice, we are with quite a large team of [executive NSCs] and everyone has their own expertise and their own eh, eh, eh, thing they get energy from. And I do notice that, as far as possible, efforts are made to give people tasks that suit them best.” [Executive NSC]

**External support**

- Quote 21: “Financial support from the municipality is necessary...what you often see in municipal processes is that they are quite slow and start slowly.” [Executive NSC]
- Quote 22: “If the municipality would focus on that and if the municipality had the vision to do more with it, um, financial support would be desirable.” [Executive NSC]
- Quote 23: “Certain choices that are made higher up and which you as an [NSC] cannot influence.” [Executive NSC]
- Quote 24: “The municipality actually thought the results were too, too small. Eh, while the investment, uh, was quite high. So eh, quite a lot of time and energy eh, went into that, but actually too little [impact], in a structural way.” [Coordinating NSC]
- Quote 25: “I am getting more and more budget....I looked for more eh, partners who eh, who have similar initiatives in the city. So uh, I looked at what we, what we can uh, do together. Eh, little by little, the more the municipality and other parties experience it, the more enthusiastic they become, the more eh, the more budget is available and the more they are convinced that it can really be an added value eh, for the city.” [Coordinating NSC]

**Financial costs**

- Quote 26: “Money is always an uh, an obstacle, let me put it this way. So, you do indeed have to have good considerations and good arguments that it has an eh, that it has added value and there is always – you always have to argue that very well and eh, eh, have that information before the choice would indeed be taken.” [Coordinating NSC]

**Time**

- Quote 27: “To shape it into an uh, a concept idea that is realistic, feasible, and that really, uh, can take place, also needs time.” [Coordinating NSC]
- Quote 28: “That will take a lot of time. Ehm, so if that threshold is lowered, it will become easier to apply it in practice.” [Executive NSC]
- Quote 29: “Of course, you have to make time to find out how it works. How are you going to apply it? But on the other hand, it also eases your work sometimes, as the app takes over a lot.” [Executive NSC]
- Quote 30: “The goal was to, uh, actually get people moving. Eh, without having to be physically present and that was eh, ideally eh, a very nice goal for us. Only in practice it turned out differently. Eh, mainly because it just takes a lot of time to prepare. Ehm, but once it’s in practice, it’s done.” [Executive NSC]
- Quote 31: “I think it’s not so much a question of do you have time, but more of, do you make time?” [Executive NSC]

#### External Support

Support from outside the organization was rarely mentioned, except for NSCs who indicated that support from external parties (eg, schools and commercial sports clubs) contributed to a more sustained implementation of gamification. In general, a strong collaboration and exchange between various stakeholders was considered important. NSCs perceived pressure to adopt and implement gamified apps when they noticed that colleagues outside their organization kept up with gamified app trends. NSCs lacked highly needed support and vision from policy makers (quotes 21 and 22, Textbox 3). Furthermore, NSCs mentioned policy makers’ power, which they could not influence (quotes 23 and 24, Textbox 3). One NSC mentioned the importance of starting small to slowly convince the municipality and other organizations and to build a sustainable gamification network (quote 25, Textbox 3). NSCs stated that it was part of their role to create sustained networks in the neighborhood. However, making external organizations enthusiastic about gamification, or providing more information about the gamification concept or the added value of gamified apps, were perceived as barriers.

#### Financial Costs

Challenges with regard to costs included (1) high product costs (eg, expensive physical gamification tools or subscriptions to gamified apps), (2) difficulties finding suitable subsidies to afford gamification tools or gamified apps, and (3) substantiating the budget plan to policy makers with persuasive arguments. Regarding high product costs, it was mentioned that it was financially challenging for smaller municipalities to subscribe to a gamified app compared to larger municipalities, as often the same price was applied and was not linked to the number of inhabitants and therefore (possible) users. In addition to subscription costs, expensive on-site products (eg, smart playgrounds) that required subsidies from municipalities were mentioned as a barrier. Furthermore, some NSCs experienced difficulty finding suitable subsidies or finding different municipal subsidies to combine, whereas others seemed to know all the ins and outs of finding the right subsidies. Justification or argumentation was important to receive the subsidy eventually. An adequate level of knowledge about gamification tools and gamified apps (eg, including scientific effects) and expected outcomes was required to justify their subsidy plans (quote 26, Textbox 3).

#### Time

For small gamification adaptations, time was not identified as an impeding factor, as this felt more intuitive for NSCs to align with their current way of working and their perceived freedom to plan their activities. However, especially preparatory work, such as justifying costs, finding scientific evidence or suitable applications, and good practices was identified as challenging (quotes 27 and 28, Textbox 3). NSCs indicated that the time investment could be worth it (quotes 29 and 30, Textbox 3). NSCs framed it as the willingness to prioritize time, which was seen as being crucial to the sustainable provision of an up-to-date app (quote 31, Textbox 3).

## Discussion

### Principal Findings

This study aimed to better understand the perceived barriers to, and facilitators of, NSCs’ use of gamification. The results reveal that NSCs have limited experience with gamification; this is in line with earlier research showing that only 4% of lifestyle professionals in the Netherlands use gamified tools [37]. In comparison with their use of gamified apps, NSCs apply gamification (without technology) more naturally in their daily work. Perceived barriers and facilitators related to skills, knowledge, expected effects, responsibility, social support, financial costs, and time. No other research has been done on NSCs’ implementation of gamification, but we can compare our results with a study focusing on the implementation of digital health tools by lifestyle professionals in the Netherlands [37]. That study found that lifestyle professionals were motivated to use digital coaching tools but required more content-specific exchanges with colleagues and a complete overview of available tools. They lacked training during their education and experienced a high financial burden when implementing digital coaching tools. All these factors align with the results of this study in terms of expected effects, knowledge, social support, skills, and financial costs [37]. Regarding responsibility and time, our findings in this study are different. Lifestyle professionals perceived the successful implementation of digital coaching as time-intensive, and hours spent could not be claimed from health insurance. In comparison, the NSCs in this study did not consider time to be a barrier. Rather, their perceived freedom in their work contributed to implementing gamification. However, both executive and coordinating NSCs felt uncertain about which of the 2 should take responsibility for implementing gamification, whereas this did not appear as a barrier for lifestyle professionals [37].

Former research among intermediaries, such as physiotherapists, physicians, and nurse practitioners, investigated the implementation of health interventions in primary care. Structurally implementing existing health guidelines (ie, PA guidelines), exercise referral schemes, or interventions (ie, mobile health, eHealth, and combined lifestyle interventions) in organizational structures and regulations were identified as a common barrier to successful implementation [30,37-44]. Besides NSCs, school teachers are important intermediaries and implementers of gamification to promote PA. They already have experience with implementing gamification with the aim of promoting PA in schools [45,46]. Schools are often considered as a place where a diverse population, including students that are often considered hard to reach, can be addressed in a structural manner (eg, integration in the curriculum). This is the first study to focus on the implementation of gamification by NSCs as intermediaries. Offering interventions via intermediaries may contribute to the successful implementation of interventions. Intermediaries can reach populations that may benefit the most from the interventions and are often considered hard to reach. Our research reveals that NSCs perceive advantages in implementing gamification (ie, expected effects) for hard-to-reach populations that are often known to engage in lower levels of PA, to reach people from a distance, and to lower the threshold to engage in their program. Expected effects emerged as one of the most frequently mentioned themes in our study. Furthermore, NSCs perceive that they have the freedom to choose suitable activities themselves and are in charge of allocating their time to a range of diverse activities. This suggests that NSCs form a specific group of intermediaries that hold promise to integrate gamification in their current work.

Coordinating and executive NSCs’ roles and responsibilities regarding the adoption and implementation of gamification appear to be unclear; this emerged as a frequently mentioned theme. This is in line with earlier research indicating that a blueprint for NSCs’ function is not in place [47] and at the same time not desired, as the freedom to implement PA activities and allocate their time independently is part of the function description [48]. In general, both coordinating and executive NSCs see gamification implementation as part of their function but experience difficulties in successfully establishing collaborations with other parties to structurally implement gamification. Moreover, a clear vision on the part of municipal policy makers could facilitate the structural implementation of gamification. As a result, strategic implementation at a systemic level is often lacking [24]. A responsible person who is enthusiastically involved and reinforces colleagues often seems to be lacking, although such a person is considered crucial for the strategic implementation of gamification in the long term to prevent NSCs from implementing gamification only sporadically. Clear assignments by the manager or municipal policy makers may support successful implementation in the future [43,44,49].

Research has shown that scaling up digital health innovations is often hindered by barriers, such as a lack of evidence or long-term financial funding, and therefore often depends on the acceptance of different individual stakeholders working in different domains or different levels of systems. Research suggests that a higher level of coordination and knowledge sharing across digital health projects is needed [50]. A promising pathway to facilitate scaling up is to incorporate systems thinking in the implementation process. Systems thinking helps to elucidate how situations and experiences are connected and influence implementations. Viewing gamification through the conceptual lens of systems thinking is deemed to have the potential to create harmonized solutions that are relevant to individuals and take the various mutually influencing levels of the system into account [51]. Systems thinking is in line with our study, as different levels in the system, such as research, policy, and practice, do not seem to align well during the implementation phase. For example, policy needs to be informed by researchers to support the scientific effects of gamification (eg, on health outcomes). In contrast, practice (eg, NSCs) lacks subsidization and policy makers’ vision. A better alignment among the 3 different levels of the system together with end users is crucial [52] to ensure adoption, implementation, and sustainability and therefore effects on health outcomes (ie, increased PA). Implementation science can facilitate this need to translate research into practice and contribute to system change. Including different stakeholders from different system levels through the development of the gamification tool is crucial. Integrating a horizontal systems approach (ie, sports clubs, schools and PA organizations) with a vertical systems approach (ie, local-, regional-, national-level governments) may strengthen synergetic effects among a varied group of stakeholders and further strengthen sustainable implementation and even scale up of gamification and gamified apps [53,54].

### Strengths and Limitations

Several limitations of this research should be considered. First, no generalizations can be made beyond the study sample. NSCs seemed to focus on children and adolescents during the interviews, although this age group was not the sole focus of this study. Possibly, NSCs relate the concept of gamification and gaming to a younger age population. As we recruited via a contact person in each organization, it might also be possible that the contact person made a preselection beforehand. According to the national NSC monitor, many NSCs do focus on children or adolescents, therefore our sample seems to be in line with the general NSC profile [55]. In addition, as the NSCs’ job description varies between municipalities, it is difficult to determine whether our sample reflects the diversity of NSC profiles currently present across municipalities. In the Netherlands, municipalities can employ executive or coordinating NSCs, or NSCs that fulfill both functions. However, some municipalities employ only coordinating NSCs or only executive NSCs, who may either lack vision, usually developed by coordinating NSCs, or lack executive activities performed by executive NSCs [48]. In our study, the interviewed NSCs in the 5 municipalities are all employed as either coordinating or executive NSCs.

Several strengths of this study can be identified too. We conducted a substantial number of qualitative interviews that led to data saturation. In addition, in terms of method, we managed to inform our analysis based on well-established theoretical models, the COM-B and TDF, that cover the wide spectrum of behavioral determinants. Coding the interviews according to the COM-B model and TDF shows that both are relevant in implementation science and can identify factors facilitating or hindering the implementation of innovation [30,38-41,56]. Moreover, the analysis was performed by 2 coders, thereby further strengthening the credibility of the study. In terms of gamification experience, we reached a diverse sample including nonexperienced, inexperienced, and experienced gamification NSCs. As this was the first study to focus on intermediaries promoting PA in the neighborhood via gamification in their daily work, we managed to contribute to a novel and relevant scientific and societal topic.

### Implications

#### For Practice

By linking the identified facilitators and barriers to the behavior change wheel [29], we can identify several relevant intervention functions, such as education (eg, providing information), training (eg, practicing technical and creative skills in workshops), modeling (eg, providing best practices and clear implementation guidelines), and environmental restructuring (eg, support a restructuring of the organization where NSCs are employed). From the results, we formulated 5 recommendations for policy makers, industry, intermediaries, and professionals (Textbox 4).

First, NSCs indicated that they lacked not only sufficient skills training to implement gamification but also education about the scientific effects and an overview of the different tools. Hence, it is recommended to provide a clear overview of tools and best practices, for example, via education and workshops. Second, NSCs felt supported by their employer who granted them a lot of freedom to design activities in line with local needs and wishes. However, NSCs indicated a lack of exchange with colleagues and external parties. A network within but also outside the organization or municipality appears to be important in terms of sharing content, best practices, and keeping up with the latest trends and developments. This relates to intersectoral collaborations [37,43,44,47,57], which might be needed among NSCs in the Netherlands to learn how to better collaborate with other parties. Here, NSCs’ professional organizations could play an important role in facilitating a network [37]. Third, coordinating and executive NSCs were uncertain about their responsibility to adopt gamification, a frequently mentioned theme. Clarifying NSCs’ responsibility, as indicated by NSCs during the interviews, for example, by appointing a dedicated gamification NSC to provide a clear assignment to executive NSCs or to allocate hours to NSCs, may help the sustainable implementation of gamification. Fourth, NSCs perceive large barriers with regard to financial costs, as product costs are high and suitable subsidies are lacking. A clear overview of subsidy options may help to better connect and scale up initiatives and integrate gamification and gamified apps sustainably. Fifth, NSCs indicated that they lacked sufficient promotion skills that they considered necessary for the successful implementation of gamification and gamified apps. Supporting NSCs to successfully embed the gamification tool in a larger promotion campaign might impact the implementation of an intervention.

**Actions that should be taken to enable all relevant stakeholders, policy makers, industry, intermediaries, and professionals to exploit the potential of gamification.**

**Recommendations**

- Establish a sustainable *network* of organizations to strengthen the offer within one municipality and to exchange ideas and best practices beyond municipalities
- Clarify the *responsibility* of neighborhood sport coaches (NSCs), for example, appoint a dedicated NSC to provide a clear assignment or to allocate hours
- Provide a clear *subsidy* overview that helps NSCs to connect initiatives and integrate gamification and gamified apps sustainably
- Offer guidance on how to *promote* gamification and gamified apps among the target group to secure book long-lasting engagement

#### For Research

One of the barriers identified by intermediaries was the lack of a clear overview of gamification that has proven effective. Research is often struggling with conclusive results on the effects of gamification and often lacks rigorous study designs [13,22,58]. A white paper that translates research results in a comprehensive manner could support intermediaries in the future to understand and refer to the proven effects, for example, to justify financial subsidies. Furthermore, more quantitative research is necessary to identify the determinants that significantly explain NSCs’ implementation of gamification across the Netherlands and how these determinants influence different stages of the implementation phase (ie, adoption, implementation, and sustainability) [59]. As differences between NSC profiles depend on the municipality, it is difficult to assess which resources contribute to the implementation of gamification, for example, small versus large municipalities’ offering. Moreover, to understand the implementation of gamification at the systems level, it is important to better align research with practice and policy, as all 3 subsystems depend on one another. Future studies may want to consider integrating the focus on different levels of the system and how they can be better aligned to implement long-lasting gamification interventions with effects on health outcomes.

### Conclusions

This study aimed to assess the barriers and facilitators that NSCs perceive toward using gamification in their daily work. NSCs highly valued the application of gamification to stimulate PA in the neighborhood. Different barriers and facilitators relating to 7 themes emerged, namely, skills, knowledge, expected effects, responsibility, social support, financial costs, and time. As this is one of the first studies to focus on NSCs that implement gamification, different practice-based and research-based implications can be considered. Providing a clear overview of tools and best practices, a clear overview of subsidies available to help NSCs connect initiatives and integrate gamification sustainably, and guidance on how to promote gamification among the target group are important actions that can be undertaken by means of written documentation. Certain restructuring policies can assist the establishment of a sustainable network of organizations to strengthen the offer within and beyond the municipality and to clarify NSCs’ responsibilities. Further research is needed to measure the effects of gamification and needs to be approached by the triangle of research, practice, and police to implement gamification in a systems approach.

## Appendix

Multimedia Appendix 1 Interview guide.

## Abbreviations

COM-B: capability, opportunity, motivation, behavior
NSC: neighborhood sport coach
PA: physical activity
TDF: theoretical domains framework
