# Supplementary material for: Perceived Barriers and Facilitators Regarding the Implementation of Gamification to Promote Physical Activity in the Neighborhood: Interview Study Among Intermediaries
Source: JMIR Serious Games. 2024 Aug 28;12:e52991. doi: 10.2196/52991 (PMC11391157; doi:10.2196/52991)
Supplement: Multimedia Appendix 1 [file games_v12i1e52991_app1.docx]

## Multimedia Appendix 1 Interview guide

1. Introduction of research project and interviewer
2. Introduction by interviewee
   1. Could you introduce yourself?
      1. What does your job entail?
      2. Which organizations do you mainly work with?
      3. Do you have a coordinating or executive role? Can you tell more about this?
      4. How much freedom do you experience in completing your work/activities?
      5. What belongs to your field of work? E.g. several municipalities, a municipality, several districts, or a specific district?
      6. Since when do you work in this position?
3. Target group
   1. On which target group do you focus?
      1. E.g. age group?
      2. E.g. socio-economic position?
      3. E.g. physical activity levels?
      4. Who has chosen to focus on this target group?
   2. Is there a target group you would like to reach, but is currently difficult to reach?
4. Definition gamification and gamified apps
   1. Are you familiar with the term gamification or gamified apps?
   2. How would you define gamification or gamified apps?
      1. Outline of definition gamification: *“improving an activity (e.g. sport or exercise) by applying game elements to make this activity (even) more fun for the user”. Think for example of scoring, challenges, rankings, competitions, badges, levels and progression bars. Gamification can be applied with or without technology.*
      2. Outline of definition gamified apps: *application and use of apps for health purposes, such as exercising more. This is really about applying technology and implies certain gamification elements.*
5. Role of gamification and gamified apps
   1. What is the current role of gamification/gamified apps in your (daily) work?
      1. If applicable:
         1. How do you apply this in your work?
         2. Can you give an example?
         3. How did you get to this point?
         4. What added value do you see from using gamification/apps?
         5. Can you tell us more about how often you use this (e.g., daily vs. occasional; explore vs. implement)?
         6. Do you collaborate with other sectors in the use of gamification/apps (e.g., youth work), if so, with whom?
         7. Do you also work with external parties (companies, organizations), and if so, with whom?
         8. What are your experiences with other sectors/external parties? (What is going well/less well)?
         9. To what extent do you experience support for the application of gamification/gamified apps from your organization?
         10. What support would you like/need?
         11. Since when do you apply this?
         12. Has the role changed recently? Think, for example, of (more or less) priority.
      2. If not applicable:
         1. Why does gamification/gamified apps have no role in your (daily) work?
         2. Are you familiar with colleagues who do apply this? What are they doing?
6. Inhibiting and promoting factors
   1. What 1. makes it challenging/ would make it challenging or 2. What helps/ would help you to apply gamification/ gamified apps in your (daily) work?
      1. For example, think of
         1. Input from others (e.g., other neighborhood sports coaches, municipality)
         2. Financial support (subsidies)
         3. Time
         4. Information and material
         5. Access to information and materials
         6. Nearby reminders
         7. Knowledge
         8. Skills
         9. Fun preparing/carrying out activities
7. Future
   1. If you had unlimited money and resources, where do you think gamification/gamified apps in your work would be in 5 years?
      1. Why do you think this?
      2. What will make this possible by then?
      3. What is needed for this?
      4. Could you explain what your ambition or the ambition of your organization is to apply gamification/apps in the future?
8. Statement
   1. “The use of gamification/ gamified apps would help to involve hard-to-reach groups of children in exercise activities in the work of neighborhood sports coaches.”
      1. Do you agree? Why (not)?
9. Final questions or remarks
   1. Do you have questions or remarks?
